# Mitofusin 1 and optic atrophy 1 shift metabolism to mitochondrial respiration during aging

**DOI:** 10.1111/acel.12649

**Published:** 2017-07-31

**Authors:** Jyung Mean Son, Ehab H. Sarsour, Anurag Kakkerla Balaraju, Jenna Fussell, Amanda L. Kalen, Brett A. Wagner, Garry R. Buettner, Prabhat C. Goswami

**Affiliations:** ^1^ Free Radical and Radiation Biology Division Department of Radiation Oncology University of Iowa Iowa City IA USA

**Keywords:** aging, metabolism, MFN1, mitochondria, OPA1, respiration

## Abstract

Replicative and chronological lifespan are two different modes of cellular aging. Chronological lifespan is defined as the duration during which quiescent normal cells retain their capacity to re‐enter the proliferative cycle. This study investigated whether changes in metabolism occur during aging of quiescent normal human fibroblasts (NHFs) and the mechanisms that regulate these changes. Bioenergetics measurements were taken in quiescent NHFs from younger (newborn, 3‐day, 5‐month, and 1‐year) and older (58‐, 61‐, 63‐, 68‐, and 70‐year) healthy donors as well as NHFs from the same individual at different ages (29, 36, and 46 years). Results show significant changes in cellular metabolism during aging of quiescent NHFs: Old NHFs exhibit a significant decrease in glycolytic flux and lactate levels, and increase in oxygen consumption rate (OCR) and ATP levels compared to young NHFs. Results from the Seahorse XF Cell Mito Stress Test show that old NHFs with a lower Bioenergetic Health Index (BHI) are more prone to oxidative stress compared to young NHFs with a higher BHI. The increase in OCR in old NHFs is associated with a shift in mitochondrial dynamics more toward fusion. Genetic knockdown of mitofusin 1 (MFN1) and optic atrophy 1 (OPA1) in old NHFs decreased OCR and shifted metabolism more toward glycolysis. Downregulation of MFN1 and OPA1 also suppressed the radiation‐induced increase in doubling time of NHFs. In summary, results show that a metabolic shift from glycolysis in young to mitochondrial respiration in old NHFs occurs during chronological lifespan, and MFN1 and OPA1 regulate this process.

## Introduction

Aging is a critical risk factor for numerous health issues and successful therapy outcome. The average global life expectancy has increased approximately 50% over the last 100 years (Vincent & Velkoff, 2010). Unfortunately, the increase in life expectancy is also a significant risk factor for various age‐related health issues (*e.g.,* cardiovascular disease, cancer, diabetes, and stroke). Therefore, additional research is needed to understand more about the basic biology of aging.

Replicative and chronological lifespans are two modes of cellular aging (Munro *et al*., 2001; Sarsour *et al*., 2005, 2012; Longo *et al*., 2012). Replicative lifespan (‘Hayflick limit’) refers to the finite number of cell divisions after which proliferating normal cells are unable to divide. Replicative lifespan is regulated by telomere shortening and mitotic attrition (Hayflick & Moorhead, 1961; Henderson & Larson, 1991). Chronological lifespan is defined as the duration of quiescence during which normal cells retain their capacity to re‐enter the proliferative cycle and exit back to quiescence (Sarsour *et al*., 2008, 2012). Chronological lifespan was first reported in yeast and later in mammalian cells (Harris *et al*., 2003; Sarsour *et al*., 2005, 2012; Fabrizio & Longo, 2007). This mode of cellular aging is independent of telomerase activity and mitotic division. Chronologically aged cells reveal a significant accumulation of reactive oxygen species (ROS) and abnormalities in mitochondrial morphology (Sarsour *et al*., 2005, 2010). Because mitochondria are the major hub of cellular metabolism and ROS production, these previously published results suggest that changes in cellular metabolism may regulate chronological lifespan.

Cellular metabolism is intimately linked to mitochondrial functions. Mitochondrial respiration, that is, oxygen consumption rate (OCR), can be used to measure mitochondrial bioenergetics (Brand & Nicholls, 2011). Recent evidence suggests that mitochondrial dynamics contributes to mitochondrial respiration (Chen *et al*., 2005; Zorzano *et al*., 2010). Mitochondrial dynamics is regulated by mitochondrial fission and fusion that influence the shape, size, and number of mitochondria (Chen & Chan, 2009). Dynamin‐related guanosine triphosphatases (GTPases) regulate mitochondrial fission and fusion events (Chan, 2012). Mitochondrial fission is regulated by dynamin‐related protein 1 (DRP1). Mitochondrial fusion is regulated by dynamin‐related GTPases mitofusin‐1 (MFN1) and mitofusin‐2 (MFN2) isoforms that are anchored in the outer mitochondrial membrane (OMM), and by optic atrophy 1 (OPA1) that is anchored in the inner mitochondrial membrane (IMM). Cells lacking MFN1 and MFN2 exhibited fragmented mitochondria, which was associated with reduced mitochondrial respiration and mitochondrial membrane potential (Chen *et al*., 2005, 2010). OPA1‐deficient cells exhibit fragmented mitochondria and reduced oxidative phosphorylation (OXPHOS; Griparic *et al*., 2004; Zanna *et al*., 2008), whereas OPA1 overexpression enhances OXPHOS (Civiletto *et al*., 2015). Alterations in mitochondrial fission proteins can also affect mitochondrial function and respiration. Dominant‐negative DRP1 mutation in mouse hepatocytes showed increases in OCR and IMM proton leak (Galloway *et al*., 2012). These previous studies suggest that mitochondrial fission and fusion significantly contribute to changes in cellular metabolism.

A link between cellular metabolism and aging has been suggested since the 1930s (reviewed in McCay *et al*., 1989); but the underlying mechanisms linking metabolic homeostasis to aging have not been fully elucidated. Results from this study show that normal human fibroblasts (NHFs) nearing the end of their chronological lifespan exhibit a metabolic shift from glycolysis to mitochondrial respiration that is associated with a lower Bioenergetic Health Index (BHI), and higher susceptibility to oxidative stress. Mitochondrial fusion proteins MFN1 and OPA1 regulate this phenomenon.

## Results

### Cellular metabolism shifts from glycolysis to mitochondrial respiration during aging of normal human fibroblasts (NHFs)

**Figure 1:**
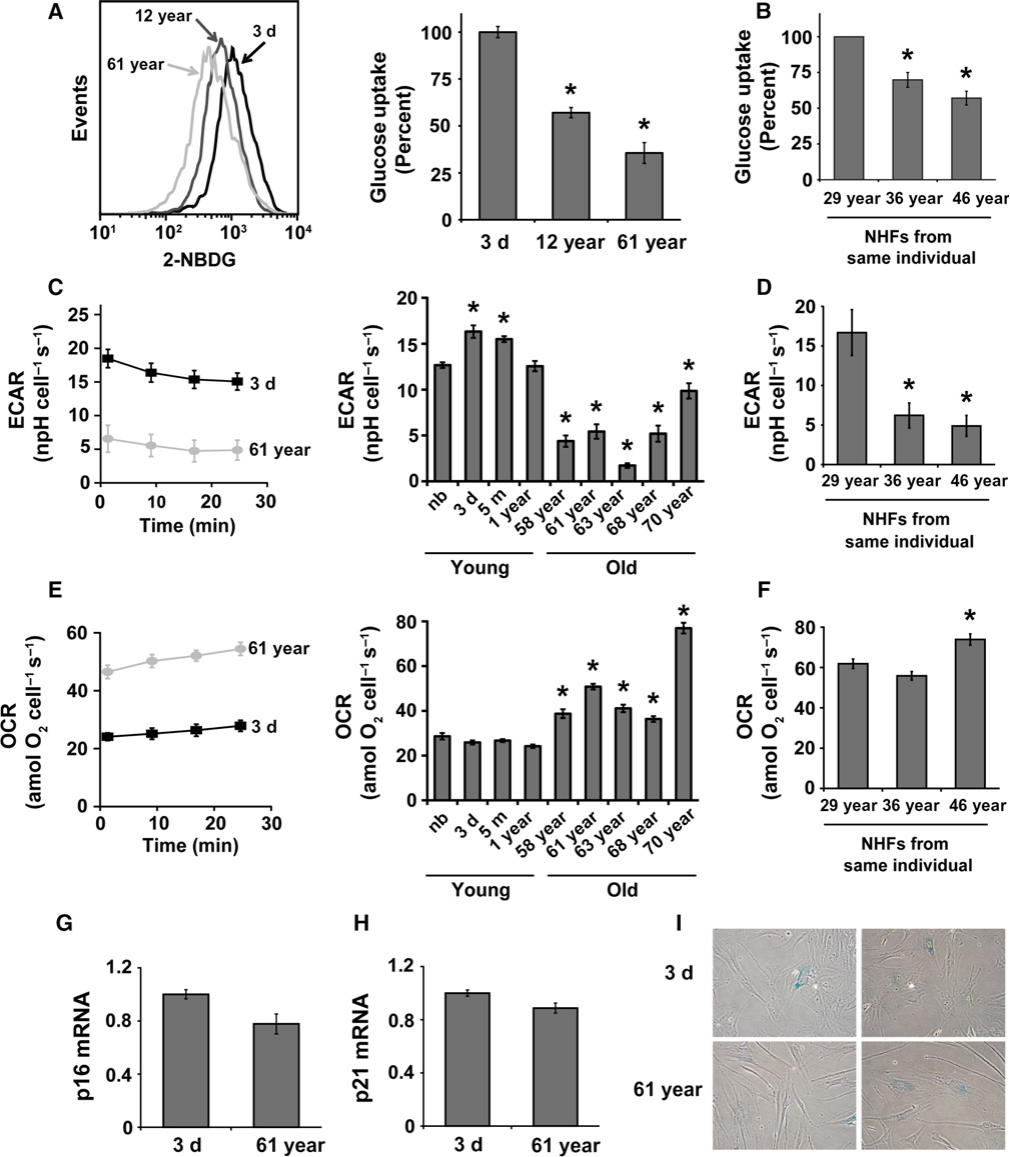
A metabolic shift from glycolysis in young to mitochondrial respiration in old quiescent normal human fibroblasts (NHFs): (A) Flow cytometry analysis of 2‐(*N*‐(7‐nitrobenz‐2‐oxa‐1,3‐diazol‐4‐yl)amino)‐2‐deoxyglucose (2‐NBDG) uptake of quiescent cultures of NHFs from healthy donors of different ages and (B) NHFs from the same individual at different ages; left panel, representative histograms; right panels, quantitation of results. Asterisks represent significance compared to 2‐NBDG uptake of (A) 3‐day NHFs and (B) 29‐year NHFs; *n *=* *3, *P *<* *0.05. (C) A Seahorse XF96 instrument was used to measure extracellular acidification rate (ECAR) in quiescent cultures of NHFs from young (newborn, 3‐day, 5‐month, and 1‐year) and old (58‐, 61‐, 63‐, 68‐, and 70‐year) healthy donors as well as (D) NHFs from the same individual at different ages: left panel, representative ECAR profile plots; right panels, quantitation of results. Asterisks represent significance compared to ECAR of (C) newborn NHFs and (D) 29‐year NHFs; *n *=* *3, *P *<* *0.05. (E) A Seahorse XF96 instrument was used to measure oxygen consumption rate (OCR) in quiescent cultures of NHFs from young and old donors as well as (F) NHFs from the same individual at different ages: left panel, representative OCR profile plots; right panels, quantitation of results. Asterisks represent significance compared to OCR of (E) newborn NHFs and (F) 29‐year NHFs; *n *=* *3, *P *<* *0.05. (G) Quantitative RT–PCR measurements of mRNA levels of p16 and (H) p21; (I) representative microscopy images of senescence‐associated β‐galactosidase activity.

The proliferative capacity of young quiescent NHFs that were aged in culture for 30–40 days was significantly suppressed (Sarsour *et al*., 2005). This loss in proliferative capacity was associated with a significant change in mitochondrial morphology and increases in cellular ROS levels (Sarsour *et al*., 2010), suggesting that cellular metabolism may regulate chronological lifespan of NHFs. To investigate this premise, a flow cytometry assay was used to measure glucose uptake in quiescent cultures of NHFs from healthy donors of different ages. Results show approximately 40% and 65% decreases in glucose uptake in 12‐ and 61‐year NHFs compared to glucose uptake in 3‐day NHFs (Fig. 1A). Consistent with these results, an age‐associated decrease in glucose uptake was also observed in NHFs from the same individual at 36 and 46 years compared to 29 years of age (Fig. 1B). An age‐related decrease in glucose uptake was also evident from the changes in the glycolytic flux. The extracellular acidification rate (ECAR), an indicator of glycolytic flux that measures pH (protons) of the extracellular medium, shows an age‐dependent decrease from 13 to 16 npH cell^−1^ s^−1^ in quiescent NHFs from younger donors (newborn, 3 day, 5 month, and 1 year) to 2–10 npH cell^−1^ s^−1^ in quiescent NHFs from older donors (58‐, 61‐, 63‐, 68‐, and 70‐year; Fig. 1C). An age‐associated decrease in ECAR was also observed in NHFs from the same individual at different ages (Fig. 1D). Consistent with the decrease in glucose uptake and glycolytic flux, a decrease in lactate levels was also observed (Fig. S1A, Supporting information). The decrease in glycolytic flux in old NHFs is associated with a significant decrease in the mRNA, protein, and activity levels of phosphofructose kinase 1 (PFK1; Fig. S2, Supporting information). These results clearly show an age‐related decrease in the glycolytic flux of NHFs.

To determine whether the decrease in glycolytic flux during aging shifts metabolism toward mitochondrial respiration, the Seahorse XF96 Analyzer was used to measure OCR. The assay measures OCR in real time; results are presented in units of attomoles O_2_ cell^−1^ s^−1^ (moles of O_2_ × 10^18^ cell^−1^ s^−1^; Wagner *et al*., 2011). Quiescent older (58‐, 61‐, 63‐, 68‐, and 70‐year) NHFs showed significant increases in OCR (36–77 amol O_2_ cell^−1^ s^−1^) compared to 24–29 amol O_2_ cell^−1^ s^−1^ in quiescent younger (newborn, 3‐day, 5‐month, and 1‐year) NHFs (Fig. 1E). An age‐related increase in OCR was also observed in NHFs from the same individual at age 46 compared to age 29 (Fig. 1F). Consistent with the increase in OCR, an increase in ATP levels was also observed in quiescent old NHFs (Fig. S1B, Supporting information). Insignificant difference in the expression of cell cycle inhibitors (p16 and p21) and β‐galactosidase activity between young and old quiescent NHFs (Fig. 1G–I) suggests that the age‐related changes in cellular metabolism may not be due to changes in cellular senescence status. An age‐related increase in OCR correlated with an increase in the oxidation of dihydroethidium (DHE; Fig. S1C, Supporting information), suggesting an increase in the steady‐state levels of cellular reactive oxygen species (ROS) during aging. An increase in the DHE oxidation during aging was associated with decreases in (i) Mn and CuZnSOD activity (Fig. S1D, Supporting information); (ii) mRNA (Fig. S1E, Supporting information) and protein (Fig. S1F, Supporting information) levels of complex II subunits; and (iii) complex II activity (Fig. S1G, Supporting information). Overall, these results indicate that a shift in metabolism from glycolysis in young to mitochondrial respiration in old NHFs is a characteristic phenomenon of chronological lifespan.

### Metabolic reprogramming and stress response during aging of NHFs

**Figure 2:**
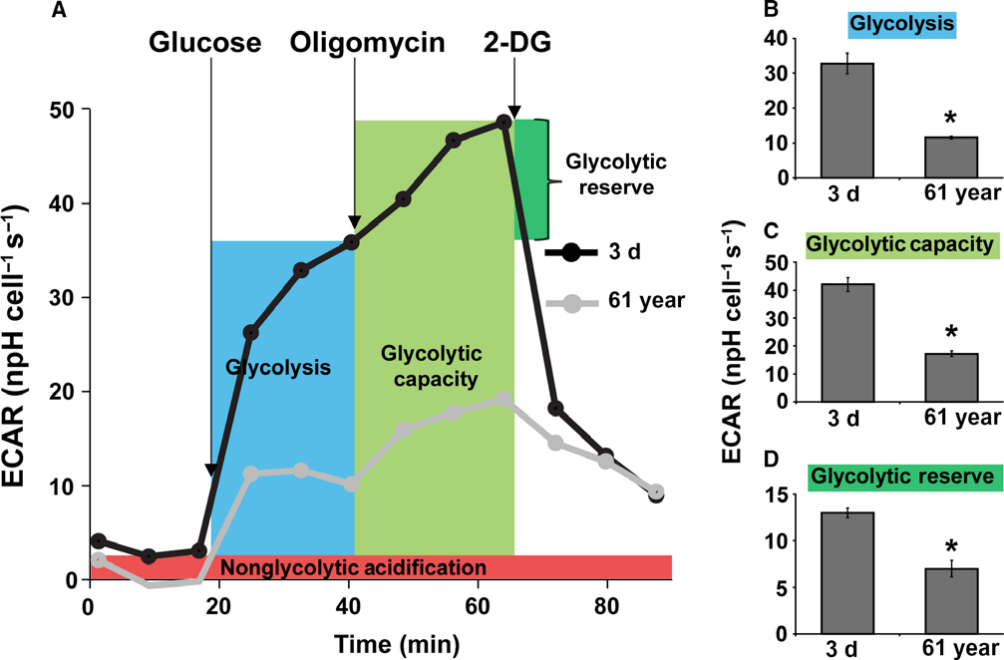
A decrease in glycolytic function during aging: Seahorse Glycolysis Stress Test was performed to measure ECAR in 3‐day and 61‐year NHFs following a sequential addition of glucose, oligomycin, and 2‐deoxyglucose (2‐DG); (A) representative ECAR profile plot, (B) glycolysis, (C) glycolytic capacity, and (D) glycolytic reserve. Basal glycolysis calculated after subtraction of nonglycolytic acidification. Glycolytic capacity was calculated following the addition of oligomycin. Glycolytic reserve was calculated based on the difference between the basal glycolysis and glycolytic capacity rates. Asterisks represent significance compared to the ECAR of 3‐day NHFs; *n *=* *3, *P *<* *0.05.

We next sought to determine whether metabolic reprogramming during aging impacts stress response of NHFs. Metabolic stress response was examined using the Seahorse XF Glycolysis and Cell Mito Stress Test kits, Seahorse Bioscience, North Billerica, MA, USA. The Glycolysis Stress Test was performed by measuring ECAR of NHFs in real time following a sequential addition of glucose, oligomycin, and 2‐deoxyglucose (2‐DG; Fig. 2). Glucose serves as a substrate for the glycolytic pathway, and the difference between ECAR before and after the addition of glucose is a measure of the glycolytic rate or glycolysis under basal conditions. Addition of oligomycin was used to block mitochondrial respiration, thereby diverting cellular metabolism toward glycolysis to meet cellular energy needs. This approach enables the measurement of the glycolytic capacity, which indicates energy production that is independent of mitochondrial respiration. 2‐DG, a glucose analog, was added to inhibit the glycolytic pathway of energy production, thereby providing a baseline measurement of ECAR. Glycolytic reserve refers to the difference between the glycolytic capacity and glycolysis. Glycolytic reserve indicates the cellular ability to increase the glycolytic rate upon increased energy demand. Three parameters of glycolytic function in real time were measured as delineated by the highlighted areas under the curve in Fig. 2A, and quantitation of results is shown in Fig. 2B–D. The poor glycolytic capacity of 61‐year NHFs is indicated by their minimal increase in ECAR following the addition of glucose (Fig. 2B), which was further evident under conditions in which respiration was blocked using oligomycin (Fig. 2C). Conversely, 3‐day NHFs were found to be highly glycolytic, which is indicated by a significant increase in ECAR following the addition of glucose (Fig. 2B) as well as the addition of oligomycin (Fig. 2C). Glycolytic reserve was also found to be significantly lower in 61‐year compared to 3‐day NHFs (Fig. 2D), suggesting that older NHFs are inefficient with the glycolytic pathway of cellular metabolism. Collectively, these results clearly demonstrate an age‐associated decrease in glycolytic flux both under basal conditions and in response to glycolytic stress.

**Figure 3:**
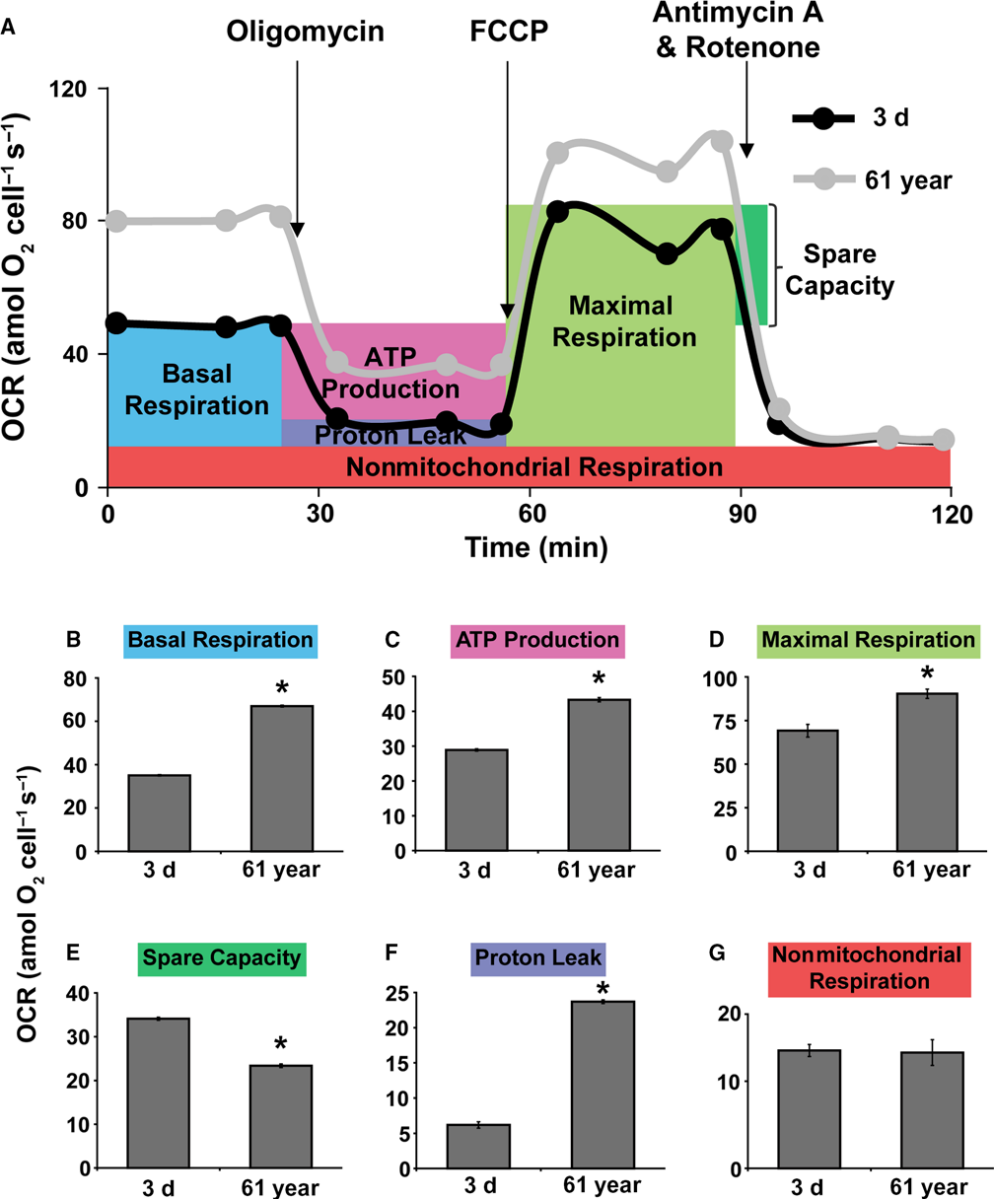
An increase in oxygen consumption rate (OCR) during aging: Seahorse Cell Mito Stress Test was performed to measure OCR in 3‐day and 61‐year NHFs following a sequential addition of inhibitors of mitochondrial function: oligomycin, carbonyl cyanide‐p‐trifluoromethoxyphenylhydrazone (FCCP), and a combination of rotenone and antimycin A; (A) OCR profile plot, (B) basal respiration, (C) ATP‐linked respiration, (D) maximal respiration, (E) spare capacity, (F) proton leak, and (G) nonmitochondrial respiration. Basal respiration was calculated after subtraction of nonmitochondrial respiration. ATP‐linked respiration and respiration of proton leak were calculated following the addition of oligomycin. Maximal respiration was measured following the addition of FCCP. Spare capacity was calculated based on the difference between the basal respiration and maximal respiration. Asterisks represent significance compared to OCR of 3‐day NHFs; *n *=* *3, *P *<* *0.05.

The Cell Mito Stress Test was used to measure cellular bioenergetics following mitochondrial metabolic stress. OCR measurements were taken in real time in 3‐day and 61‐year NHFs at basal levels and following sequential addition of mitochondrial respiration inhibitors: oligomycin, carbonyl cyanide‐p‐trifluoromethoxyphenylhydrazone (FCCP), and a combination of antimycin A and rotenone (Fig. 3). Oligomycin, an inhibitor of ATP synthase, was used to distinguish between oxygen consumption that cells use to synthesize ATP (ATP‐linked respiration) and oxygen consumption that is used to overcome the proton leak across the mitochondrial membrane (proton leak‐linked respiration). FCCP treatment collapses the proton gradient and disrupts the mitochondrial membrane potential, which allows measurements of the maximal uncoupled respiration (maximal respiration). A combination treatment of rotenone, a complex I inhibitor, and antimycin A, a complex III inhibitor, was used to shut down mitochondrial respiration, which enables differentiation between the mitochondrial (basal respiration) and nonmitochondrial (nonmitochondrial respiration) contribution to total cellular respiration. The difference between maximal and basal respiration constitutes the spare capacity. Real‐time measurements of OCR are shown in Fig. 3A, and quantitation of results is shown in Fig. 3B–G. Consistent with the results shown in Fig. 1E,F, results from the Cell Mito Stress Test also showed a significant increase in the basal respiration of quiescent old compared to young NHFs (Figs 3B and S3A, Supporting information). These results are also consistent with increases in ATP‐linked respiration (Figs 3C and S3B, Supporting information) and maximal rate of respiration in old compared to young NHFs (Fig. 3D). Respiratory spare capacity represents the reserve capacity of a cell to generate ATP *via* oxidative phosphorylation (OXPHOS) in the event of an increased demand for energy. This bioenergetics capacity of mitochondria was found to be significantly lower in 61‐year compared to 3‐day NHFs (Fig. 3E), suggesting that older NHFs are more prone to oxidative stress. This hypothesis is also supported by results demonstrating a significant increase in proton leak in old compared to young NHFs (Figs 3F and S3C, Supporting information). Both increase in proton leak and decrease in respiratory spare capacity in the old compared to the young NHFs suggest that changes in mitochondrial function do occur during aging. Results shown in Fig. 3G indicate that aging of NHFs does not impact residual respiration. Overall, these results show an increase in ATP‐linked oxygen consumption, diminished respiratory efficiency, and depletion of the respiration reserve capacity during aging of NHFs.

### MFN1 and OPA1 regulate a metabolic shift from glycolysis to mitochondrial respiration during aging

**Figure 4:**
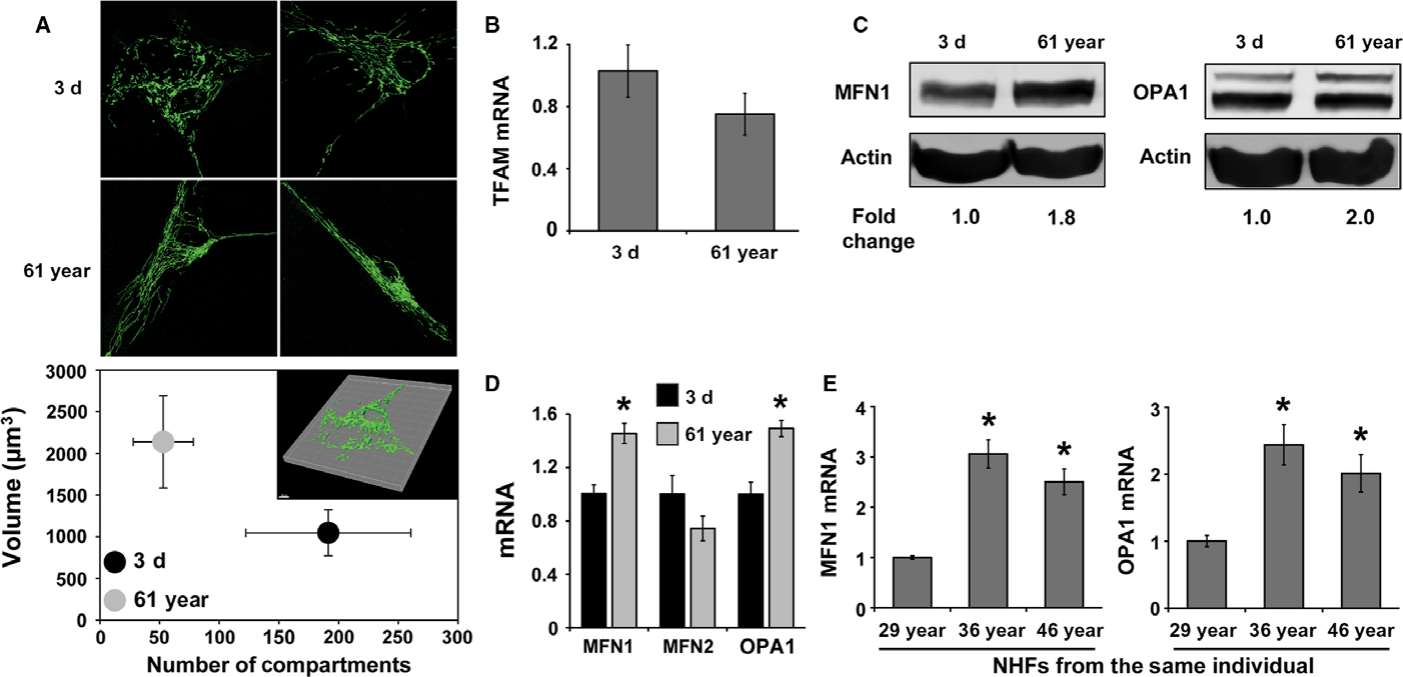
Mitochondrial dynamics shifts more toward fusion during aging: (A) confocal microscopy measurements of MitoTracker Green (Invitrogen) fluorescence in 3‐day and 61‐year NHFs (Upper panel). Imaris Scientific 3D/4D Image Processing and Analysis Software (Bitplane Scientific Software) was used to create a virtual model of the mitochondrial network (inset) as well as to calculate mitochondrial volume and number of compartments (1 μm^3^ = 1 fL). Results are plotted as the mean and standard error of the mean (lower panel); *n *=* *6, *P *<* *0.05. (B) Quantitative RT–PCR measurements of mitochondrial transcription factor A (TFAM) mRNA levels; (C) immunoblotting and (D) quantitative RT–PCR measurements of mitofusin 1 (MFN1), mitofusin 2 (MFN2), and optic atrophy 1 (OPA1) in quiescent cultures of NHFs. Asterisks represent significance compared to corresponding mRNA levels in 3‐day NHFs; *n *=* *3, *P *<* *0.05. (E) Quantitative RT–PCR measurements of MFN1 and OPA1 mRNA levels in NHFs from the same individual at different ages. Asterisks represent significance compared to corresponding mRNA levels of 29‐year NHFs; *n *=* *3, *P *<* *0.05.

Mitochondrial fission and fusion contribute to changes in mitochondrial respiration (Chen *et al*., 2005; Zorzano *et al*., 2010). To determine whether the age‐associated increase in mitochondrial respiration could be due to changes in mitochondrial fission and fusion events, confocal microscopy analysis of MitoTracker Green‐labeled NHFs was performed. Z‐stack fluorescence images of mitochondria are shown in Fig. 4A upper panel. Images were processed to create a virtual model of mitochondria (Fig. 4A lower panel inset) followed by calculation of volume and number of compartments (Fig. 4B). Results show that, with age, NHFs have fewer compartments, but a larger mitochondrial volume. The greater number of compartments is indicative of fragmented (fission) mitochondria, whereas the increase in volume is indicative of elongated and interconnected (fusion) mitochondria. These results show a higher abundance of mitochondrial fusion in older NHFs correlating with increases in OCR (Figs 1 and 3). No significant change was observed in mRNA levels of mitochondrial transcription factor A (TFAM) between 3‐day and 61‐year, suggesting that the increase in OCR of old NHFs is probably not due to a change in mitochondrial biogenesis (Fig. 4B).

In general, mitochondrial fusion is regulated by MFN1, MFN2, and OPA1. To determine whether the age‐associated increase in mitochondrial fusion could be related to changes in the expression of MFN1, MFN2, and OPA1, western blotting and quantitative RT–PCR assays were performed. Results show increases in protein and mRNA levels of MFN1 and OPA1 in 61‐year compared to 3‐day NHFs (Fig. 4C,D). OPA1 antibody detected both the short (soluble form present in mitochondrial intermembrane space) and long forms (anchored in IMM) of OPA1. It is believed that the long form is the fusion‐active form of OPA1 (Anand *et al*., 2014), which was found to be increased in 61‐year NHFs. Consistent with these results, a significant increase in MFN1 and OPA1 mRNA levels was also observed in NHFs at age 36 and age 46 compared to age 29 of the same individual (Fig. 4E), suggesting that the age‐related increase in mitochondrial fusion gene expression is a true phenomenon of aging.

**Figure 5:**
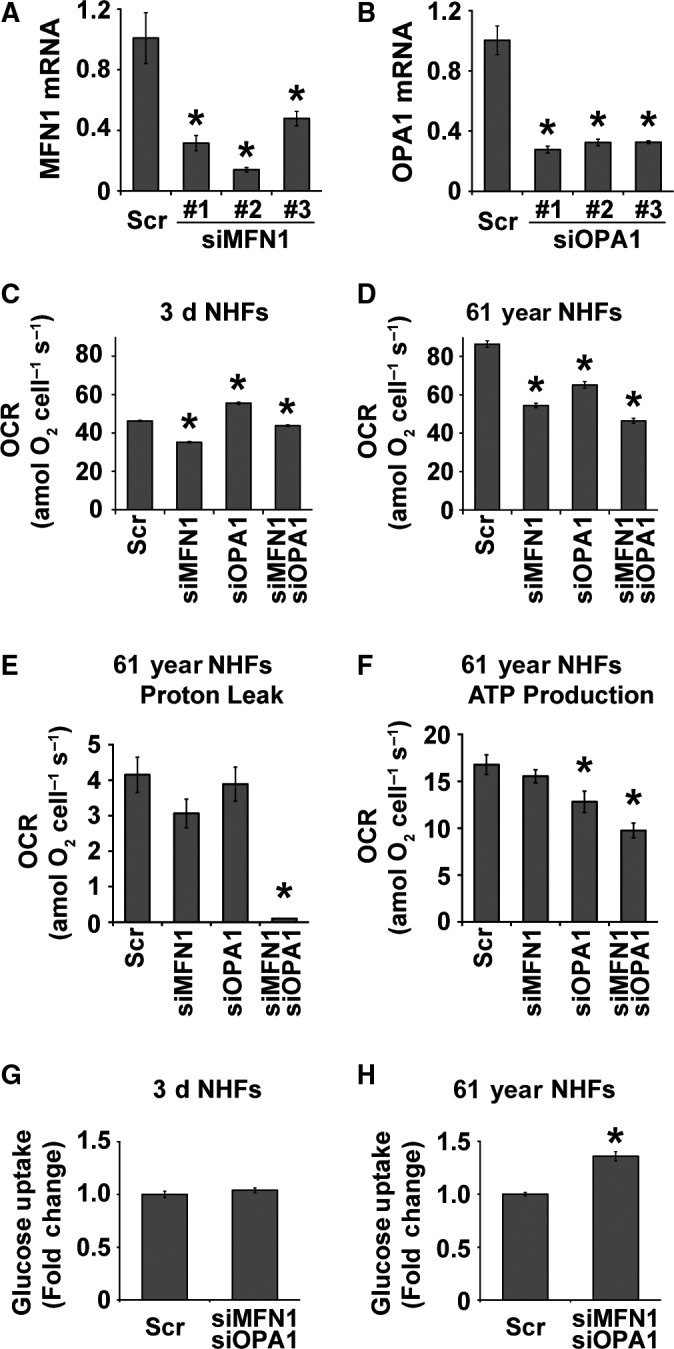
MFN1 and OPA1 regulate mitochondrial respiration during aging: quantitative RT–PCR measurements of (A) MFN1 and (B) OPA1 mRNA levels in scrambled siRNA (Scr)‐, MFN1 siRNA‐1, ‐2, ‐3‐, or OPA1 siRNA‐1, ‐2, ‐3‐treated quiescent NHFs. Quiescent NHFs were transfected with either Scr, MFN1 siRNA‐2, OPA1 siRNA‐1, or the combination of MFN1 and OPA1 siRNAs and metabolic endpoints were measured at 48 h post‐transfection: the Seahorse XF96 measurements of OCR (C and D), proton leak (E), and ATP‐linked OCR (F); and flow cytometry measurements of 2‐NBDG uptake (G and H). Asterisks represent significance compared to Scr‐treated NHFs; *n *=* *3, *P *<* *0.05.

To determine the causality of MFN1 and OPA1 regulating mitochondrial respiration during aging, RNA interference approach was applied to knockdown expression of MFN1 and OPA1, and then, OCR was measured. All three MFN1 and OPA1 siRNA constructs decreased MFN1 and OPA1 mRNA levels, respectively, by more than 50% (Fig. 5A,B). Based on the knockdown results, siMFN1 #2 and siOPA1 #1 were selected for further studies. NHFs were transfected with Scr, MFN1, OPA1, and combination of MFN1 and OPA1 siRNAs followed by measurements of OCR. OCR of quiescent 3‐day NHFs decreased approximately 24% in siMFN1‐treated cells, whereas treatment with siOPA1 showed approximately 20% increase in OCR (Fig. 5C). The combination of siMFN1 and siOPA1 treatment showed only a minimal decrease in OCR of 3‐day NHFs. Surprisingly, downregulation of MFN1 and OPA1 in quiescent 61‐year NHFs showed a dramatic response (Fig. 5D): approximately 37% decrease in OCR of siMFN1‐transfected cells, 24% decrease in OCR of siOPA1‐transfected cells, and 46% decrease in OCR of siMFN1‐siOPA1‐transfected cells. Furthermore, results from the Cell Mito Stress Test showed that downregulation of MFN1 and OPA1 expression in the 61‐year NHFs significantly decreased proton leak and ATP‐linked respiration compared to the scrambled siRNA‐transfected cells (Fig. 5E,F). It is interesting to note that the decrease in mitochondrial respiration in 61‐year NHFs transfected with a combination of siMFN1 and siOPA1 shifted metabolism more toward glycolysis, whereas glucose uptake in 3‐day NHFs showed no significant difference (Fig. 5G,H). These results demonstrate that a shift toward mitochondrial fusion during aging is associated with increases in mitochondrial respiration; MFN1 and OPA1 regulate this process.

### Old NHFs with a lower Bioenergetic Health Index (BHI) are more prone to oxidative stress

**Figure 6:**
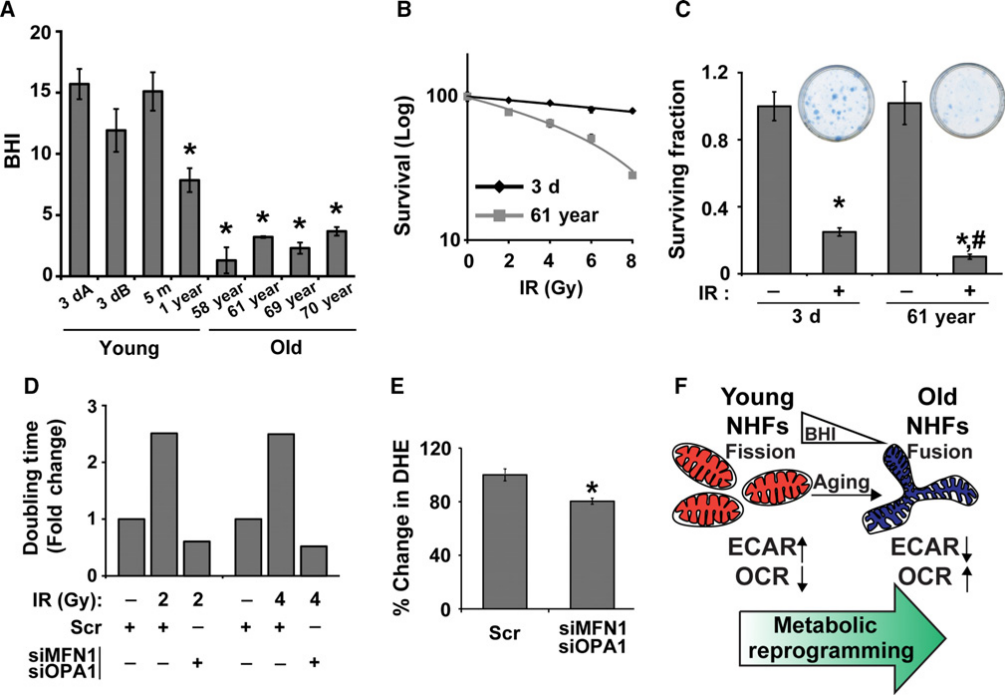
Older NHFs with a lower Bioenergetic Health Index (BHI) are more prone to oxidative stress. (A) Results from the Seahorse Cell Mito Stress Test were used to calculate BHI as follows: (reserve capacity × ATP‐linked OCR)/(proton leak OCR × nonmitochondria OCR; Chacko *et al*., 2014). 3‐dayA and 3‐dayB are NHFs from two different 3‐day healthy donors. Asterisks represent significance compared to the BHI of 3‐dayA NHFs; *n *=* *3, *P *<* *0.05. (B) A flow cytometry‐based propidium iodide (PI) exclusion assay and (C) a clonogenic assay were used to measure ionizing radiation‐induced toxicity of quiescent young and old NHFs. Inset shows representative dishes with colonies from the clonogenic assay. Asterisks represent significance compared to un‐irradiated control cells; # represents significance compared to irradiated 3‐day NHFs; *n *=* *3, *P *<* *0.05. (D) Cell doubling time was measured in Scr‐ and siMFN1‐siOPA1‐treated and irradiated 61‐year NHFs; fold change in doubling time was calculated relative to un‐irradiated Scr‐treated cells. (E) Flow cytometry measurements of DHE oxidation in 8 Gy irradiated Scr‐ and siMFN1‐siOPA1‐transfected 61‐year NHFs at 48h postirradiation. Percent change in DHE oxidation was calculated in irradiated cells relative to their corresponding un‐irradiated cells. Asterisks represent significance compared to Scr‐transfected irradiated cells; *n *=* *3, *P *<* *0.05. (F) An illustration showing a shift in mitochondrial dynamics from fission in younger to fusion in older NHFs contributing to the metabolic reprogramming from glycolysis in young to mitochondrial respiration in old NHFs.

Mitochondria respond to the changes in their cellular environment. Results from the metabolic stress test (Figs 2 and 3) suggest that older NHFs are more susceptible to stress. To further investigate this premise, initially, we measured BHI of NHFs from different ages. BHI represents a dynamic index of oxidative stress response that serves as a single integrated value of the individual parameters of the bioenergetics function in the cell (Chacko *et al*., 2014). BHI is calculated as a ratio of OCR of spare capacity (Fig. 3E) and ATP‐linked OCR (Fig. 3C) to OCR of proton leak (Fig. 3F) and nonmitochondrial OCR (Fig. 3G). BHI was found to be significantly lower in the older NHFs (58‐, 61‐, 69‐, and 70‐year) and higher in the younger NHFs (3‐dayA, 3‐dayB, 5‐month, and 1‐year; Fig. 6A). Cells with a higher BHI are anticipated to adapt better to oxidative stress, whereas cells with a lower BHI are anticipated to be more prone to oxidative stress. Indeed, results obtained from a flow cytometry assay of propidium iodide (PI)‐positive (representing nonviable cells) and PI‐negative (representing viable cells) population of cells showed a dose‐dependent radiation‐induced toxicity for both 3‐day and 61‐year NHFs (Fig. 6B). However, 61‐year NHFs with a lower BHI were found to be significantly more sensitive to radiation treatment compared to 3‐day NHFs with a higher BHI. The lethal dose that results in 50% of the PI‐positive cells was only 6 Gy in 61‐year NHFs compared to 18 Gy in 3‐day NHFs. These results are comparable with the results obtained from a clonogenic assay (Fig. 6C). The surviving fraction in 2 Gy‐irradiated 3‐day young NHFs was 0.3, whereas the 61‐year NHFs showed a significantly lower surviving fraction of 0.1. A link among OCR (Figs 1 and 3), mitochondrial fusion (Fig. 4), and stress response (Fig. 6A–C) was also found in irradiated siMFN1‐ and siOPA1‐transfected 61‐year NHFs (Fig. 6D,E). As anticipated, radiation treatment resulted in a significant increase in cell doubling time in Scr‐treated 61‐year NHFs. Interestingly, this increase in doubling time was suppressed in siMFN1‐ and siOPA1‐transfected 61‐year NHFs (Fig. 6D). Results from flow cytometry measurements of DHE oxidation showed that cellular ROS levels were significantly lower in siMFN1‐ and siOPA1‐transfected 61‐year NHFs (Fig. 6E). Overall, these results demonstrate that BHI is a biomarker for stress response; older NHFs with a lower BHI are more prone to oxidative stress.

In summary, results from this study show that mitochondrial fusion is associated with a shift from glycolysis in young to mitochondrial respiration in old NHFs. Old NHFs with a lower BHI are more prone to oxidative stress. MFN1 and OPA1 regulate both of these processes (Fig. 6F).

## Discussion

Chronological lifespan and replicative lifespan are two types of cellular aging (Munro *et al*., 2001; Sarsour *et al*., 2005, 2012; Longo *et al*., 2012). Our earlier results (Sarsour *et al*., 2010, 2012) and that mitochondria being the hub of cellular metabolism led us to investigate whether changes in cellular metabolism can regulate chronological lifespan. Results showed that cellular metabolism shifts more toward mitochondrial respiration during aging. An age‐related increase in mitochondrial respiration negatively impacts NHFs’ ability to cope with oxidative stress. Mitochondrial fusion proteins, MFN1 and OPA1 regulate both of these processes: mitochondrial respiration and response to oxidative stress.

Metabolic dysfunctions during aging have been observed in lower eukaryotes and invertebrates. Metabolic changes have been reported for both replicative and chronological lifespan of yeasts (Longo *et al*., 2012). In *Caenorhabditis elegans*, metabolic activity and oxygen consumption of wild‐type worms change with aging (Collins *et al*., 2008). *Drosophila melanogaster* exhibits age‐related downregulation of numerous mitochondrial genes (Landis *et al*., 2012). Metabolic changes during aging of vertebrate animals have also been reported (Riera & Dillin, 2015). Lapointe and Hekimi showed that mouse lifespan is linked to mitochondrial function including mitochondrial coupled respiration and ATP synthesis (Lapointe & Hekimi, 2008). While these previous studies indicate metabolic changes during aging in lower eukaryotes, invertebrates, and mice, metabolic changes during aging of humans are not completely delineated.

In this study, we used quiescent cultures of NHFs from healthy donors of different ages, and measured glycolytic flux and mitochondrial respiration. Results from flow cytometry measurements of 2‐(*N*‐(7‐nitrobenz‐2‐oxa‐1,3‐diazol‐4‐yl)amino)‐2‐deoxyglucose (2‐NBDG) uptake (Fig. 1A) and Seahorse analysis of ECAR (Fig. 1C) show a significant decrease in glucose uptake and ECAR in the old compared to the young NHFs. The decrease in ECAR in old NHFs correlates with a decrease in lactate levels (Fig. S1A, Supporting information). An age‐related decrease in glycolytic flux is associated with a significant decrease in PFK1 expression and activity; PFK1 governs the rate‐limiting step of glycolysis (Fig. S2, Supporting information). Interestingly, OCR (Fig. 1E) and intracellular ATP levels (Fig. S1B, Supporting information) were found to be significantly higher in the old compared to young NHFs. Comparable results were also observed in quiescent NHFs from the same individual at different ages (Fig. 1B,D,F). A shift in cellular metabolism from glycolysis in young to mitochondrial respiration in old NHFs is not due to a change in cellular senescence status because there were no significant differences in the expression of p16 and p21 cell cycle inhibitors as well as activity of β‐galactosidase between young and old NHFs (Fig. 1G–I). However, an age‐related increase in cellular ROS levels and decrease in SOD and complex II activity were observed in old NHFs (Fig. S1C–G, Supporting information). These results suggest that a metabolic shift from glycolysis in young to mitochondrial respiration in old NHFs is a true phenomenon of aging that appears to be independent of cellular senescence status, but dependent on mitochondrial functions.

Although an increase in OCR and ATP levels (Figs 1, 3 and S3, Supporting information) was observed in old NHFs, they also exhibited an increase in the steady‐state levels of ROS and a decrease in complex II and SOD activities (Fig. S1, Supporting information). These results suggest that the increase in OCR and ATP levels in old NHFs could be related to their response to oxidative stress. Oxidative stress can affect mitochondrial functions, for example, ATP‐linked respiration, reserve capacity, and proton leak. Indeed, results from the Seahorse Glycolytic and Mito Stress Tests did show a significant decrease in the glycolytic capacity and glycolytic reserve (Fig. 2), reduction in spare capacity, and an increase in ATP‐linked respiration and proton leak (Figs 3 and S3, Supporting information) in old compared to young NHFs. These results are also consistent with a lower BHI in old compared to young NHFs (Fig. 6A). Old NHFs with a lower BHI are more sensitive to radiation‐induced toxicity compared to young NHFs with a higher BHI (Fig. 6B,C), suggesting that mitochondrial functions contribute to oxidative stress response of NHFs during aging. Both suppression and stimulation of mitochondrial functions during aging have been reported for yeast, *C. elegans*,* Drosophila*, and mice (Marzetti *et al*., 2013; Siegel *et al*., 2013). Suppression of mitochondrial respiration extends lifespan of *C. elegans* (Dillin *et al*., 2002; Lee *et al*., 2003). These previous reports and results presented in this study suggest that inefficiency in mitochondrial functions contributes to the aging process and oxidative stress may account for an increase in OCR and ATP levels of old NHFs. Indeed, suppression of oxidative stress has been shown earlier to extend chronological lifespan of NHFs (Sarsour *et al*., 2012).

Changes in mitochondrial functions during aging can impact their morphology. Mitochondrial dynamics appears to be at the crossroads of the aging process. The frequency of mitochondrial fusion is higher in old NHFs, while young NHFs exhibit more mitochondrial fission (Fig. 4). These results suggest that mitochondrial fusion that provides more surface area may account for the increases in OCR and ATP levels observed in old NHFs (Figs 1, 3, and S3, Supporting information). Our results are consistent with earlier reports of an age‐dependent increase in mitochondrial size (fusion) in aged *C. elegans* (Yasuda *et al*., 2006); senescent human liver cells (Lee *et al*., 2007); and human skeletal muscle cells from the elderly (Beregi & Regius, 1987). While these previous studies and results presented here clearly showed a higher frequency of mitochondrial fusion during aging, other studies report a higher abundance of mitochondrial fragmentation (fission) in aged neurons of *C. elegans* (Jiang *et al*., 2015) and *in vitro* aged rat muscle cells (Iqbal *et al*., 2013). Genetic manipulation of fission and fusion machinery perturbs lifespan of yeast (Bernhardt *et al*., 2015); *Drosophila* (McQuibban *et al*., 2006); and *C. elegans* (Yang *et al*., 2011). Overall, these previous reports support a role for the mitochondrial fusion and fission regulating lifespan. Our results show that mitochondrial fission is more abundant in young NHFs correlating with lower OCR and ATP levels, whereas a higher frequency of mitochondrial fusion is associated with higher OCR and ATP levels in old NHFs.

The molecular mechanisms regulating mitochondrial fusion are complex. Increases in MFN1 and OPA1 expression are associated with a significant increase in mitochondrial fusion (Fig. 4), which correlates with increases in OCR and ATP levels of old NHFs (Figs 1, 3, and S3, Supporting information). siRNA‐mediated knockdown of MFN1 and OPA1 resulted in a significant decrease in OCR and an increase in glucose uptake in old NHFs (Fig. 5D,H). Furthermore, siRNA‐mediated downregulation of MFN1 and OPA1 expression significantly suppressed an age‐related increase in proton leak and ATP levels of old NHFs (Fig. 5E,F). In addition, knockdown of MFN1 and OPA1 suppressed radiation‐induced increase in cellular ROS levels and delays in cell doubling time of old NHFs (Fig. 6D,E). These results are consistent with earlier reports of depletion of MFNs or OPA1 modifying cellular metabolism that includes significant reductions in OCR and mitochondrial proton leak in mouse fibroblasts and myoblasts (Bach *et al*., 2003; Chen *et al*., 2005). Furthermore, increases in MFNs and OPA1 expression have been reported for replicative senescence of human hepatoma cell and mink lung epithelial cells (Lee *et al*., 2007; Park *et al*., 2010). Enhancement of MFN1 activity by S3‐mediated inhibition of USP30 (mitochondria‐localized deubiquitinase) restored mitochondrial morphology and ATP levels in fusion‐deficient mouse embryo fibroblasts (Yue *et al*., 2014). These previous studies suggest that MFNs and OPA1 regulate mitochondrial fusion and functions. Results from this study show that MFN1‐ and OPA1‐mediated regulation of mitochondrial fusion contributes to increases in OCR and ATP levels during aging of NHFs and their response to oxidative stress (Fig. 6F).

In summary, results from this study show that metabolic reprogramming from glycolysis in young to mitochondrial respiration in old NHFs is a universal phenomenon of chronological lifespan, and mitochondrial fusion proteins MFN1 and OPA1 regulate this process. Intervention of metabolic reprogramming during aging may extend healthy lifespan and minimize age‐related health issues.

## Experimental procedures

Additional details of the methods are included in the Data S1 (Supporting information).

### Cell culture

Human normal dermal fibroblasts (NHFs) were obtained from the Coriell Cell Repositories and cultured following the supplier's protocol. Quiescent cultures of NHFs were irradiated (dose rate: 0.65 Gy min^−1^) using a ^137^Cs source (JL Shepherd).

### Glucose uptake assay

BD LSRII cytometer (BD Biosciences, San Jose, CA, USA) was used to measure 2‐NBDG (Invitrogen, Carlsbad, CA, USA) uptake in quiescent cultures of NHFs.

### Metabolic flux analysis

ECAR and OCR were measured using the Seahorse XF96 Extracellular Flux Analyzer (Seahorse Bioscience, North Billerica, MA, USA). Seahorse XF assay media supplemented with 2 mm Glutamax was used in the Glycolytic Stress Test. The Cell Mito Stress Test media supplemented with 2 mm Glutamax, 1 mm sodium pyruvate, and 25 mm glucose was used.

### Imaging and quantitative analysis of mitochondrial dynamics

Quiescent cultures of NHFs were incubated with MitoTracker Green (Invitrogen), and fluorescence was visualized by a Zeiss 510 confocal microscope (Carl Zeiss, Oberkochen, Germany).

### Cell survival assays

Cell survival was examined using flow cytometry‐based measurements of propidium iodide exclusion and clonogenic assays.

### cDNA synthesis and quantitative Real‐time PCR

Total cellular RNA was extracted and the cDNA was synthesized using High Capacity cDNA Reverse Transcription Kit (Applied Biosystems, Foster City, CA, USA). Real‐time PCR amplification was performed using primer pairs specific for target genes. Relative mRNA levels were calculated: Δ*C*_T_ (sample) = *C*_T_ (mRNA of interest)—*C*_T_ (18S); relative expression = $2^{-\Delta \Delta C_{T}}$.

### siRNA knockdown

Human MFN1 and OPA1 siRNAs (OriGene, Rockville, MD, USA) were used to downregulate MFN1 and OPA1 expression.

### Immunoblotting assay

Immunoblotting was performed using antibodies to human MFN1 (Abcam, Cambridge, United Kingdom), OPA1 (BD Biosciences), and actin (Millipore).

### Senescence assay

Senescence‐associated β‐galactosidase Activity Assay (BioVision Technologies, Milpitas, CA, USA) was performed to measure cellular senescence. Senescence status was also examined by measuring mRNA expression of cyclin‐dependent kinase inhibitors (p16 and p21).

### Statistical analysis

Statistical analyses were performed using GraphPad Prism Software 6.0 (GraphPad Software Inc., La Jolla, CA, USA). Homogeneity of variance was assumed at 95% confidence interval.

## Author contributions

J.S. and P.C.G. formulated the concept, experimental design, and data analysis of this research project, and wrote the manuscript. J.S., E.H.S., A.K., J.F., A.L.K., and B.A.W. performed experiments and assisted P.C.G with data analysis. G.R.B assisted with the design and data analysis for the Seahorse XF96 measurements of bioenergetics.

## Funding

This work was supported by the NIH (2R01‐CA111365, CA169046, and P30 CA086862) and Holden Comprehensive Cancer Center funding.

## Conflict of interest

All authors declare no conflict of interest.

## Supporting information


**Fig. S1** An age‐related shift in metabolism from glycolysis in young to mitochondrial respiration in old NHFs is associated with: a decrease in lactate levels; an increase in ATP levels; an increase in cellular ROS levels; and a decrease in superoxide dismutase (SOD) and complex II activities.
**Fig. S2** Significant downregulation of phosphofructokinase 1 (PFK1) expression during aging.
**Fig. S3** Perturbations in mitochondrial functions during aging.
**Data S1** Experimental procedures.
