# Supplementary material for: Mitofusin 1 and optic atrophy 1 shift metabolism to mitochondrial respiration during aging
Source: Aging Cell. 2017 Jul 31;16(5):1136–45. doi: 10.1111/acel.12649 (PMC5595680; doi:10.1111/acel.12649)
Supplement: Supplementary file 1 — Fig. S1 An age‐related shift in metabolism from glycolysis in young to mitochondrial respiration in old NHFs is associated with: a decrease in lactate levels; an increase in ATP levels; an increase in cellular ROS levels; and a decrease in superoxide dismutase (SOD) and complex II activities. Fig. S2 Significant downregulation of phosphofructokinase 1 (PFK1) expression during aging. Fig. S3 Perturbations in mitochondrial functions during aging. Data S1 Experimental procedures. [file ACEL-16-1136-s001.pdf]

SUPPORTING INFORMATION

SI EXPERIMENTAL PROCEDURES

Cell culture

Normal human dermal fibroblasts (NHFs) from donors of different ages (newborn, AG014591; 3-d, AG01521 (3-dA); 3-d, AG01522 (3-dB); 5-m, GM08333; 1-y, GM05659; 12-y, AG16409; 58-y, AG07723; 61-y, AG02261; 63-y, AG09157; 68-y, AG13004; 69-y, AG13198; and 70-y, GM01681)), and NHFs from the same individual at different ages (29-y, AG04054B; 36-y, AG11557; and 46-y old, AG13442) were obtained from the Coriell Cell Repositories – Coriell Institute for Medical Research. NHFs were cultured in Dulbecco's modified Eagle's medium (Life Technologies) supplemented with 10% fetal bovine serum and antibiotics. All experiments were performed using cells from passage 3 and 4. To mimic chronological lifespan, contact-inhibited quiescent cultures were used in all experiments. Cesium-137 gamma radiation (JL Shepherd) was used to irradiate quiescent cultures of NHFs (dose rate: 0.65 Gy min^{-1}). Doubling time (Td) of NHFs was calculated using the equation: $Td = 0.693t/\ln(N_t/N_0)$, where t is time in days, and N_t and N_0 represent cell numbers at time t and initial time, respectively.

Flow cytometry assays

Dihydroethidium (DHE) oxidation: Monolayer cultures were incubated with Hanks buffer salt solution (HBSS) containing 10 µM DHE (Invitrogen) for 45 min. The flow cytometry measurements of DHE-fluorescence were performed using 488 nm excitation laser and 585 nm band pass emission filter. Data from 10,000 events were collected in List mode and mean fluorescence intensity was analyzed using Flowjo software (Flowjo, LLC). Auto-fluorescence of unlabeled cells was used for background fluorescence correction.

Propidium iodide (PI) exclusion assay: Control and irradiated quiescent cultures of NHFs were re-suspended in PBS and incubated with PI (1 µg mL^{-1}). PI fluorescence (excitation 488 nm, emission 585 nm) was measured using a FACScan flow cytometer (Becton–Dickinson). Data from 10,000 events were collected and population of PI-positive (nonviable) and negative (viable) cells were calculated using WinMDI software (Scripps Research Institute).

Glucose uptake: Contact inhibited quiescent cultures of NHFs were incubated with DMEM containing glucose (1 g L^{-1}) and 2-(*N*-(7-Nitrobenz-2-oxa-1,3-diazol-4-yl)Amino)-2-Deoxyglucose) (2-NBDG: 20 µM) for 2 h at 37 °C. Cells were collected by trypsinization and resuspended in PBS. 2-NBDG uptake was measured by using a BD LSRII cytometer (excitation at 465 nm, emission at 540 nm). The mean fluorescence intensity (MFI) of 20,000 cells was analyzed for each sample and corrected for autofluorescence.

Quantitative RT-PCR assay

Total cellular RNA was extracted using TRIzol reagent (Invitrogen) and quantified using a NanoDrop-1000 Spectrophotometer (Thermo Fisher Scientific). One microgram of total RNA

was reverse-transcribed using High Capacity cDNA Reverse Transcription Kit (Applied Biosystems) run on a PTC 200 Thermocycler (MJ Research): 25 °C for 10 min; 37 °C for 120 min; 85 °C for 5 min. Two microliters of cDNA was subjected to Real Time PCR amplification using the Power SYBR Green Master Mix (Applied Biosystems) and StepOne Plus™ System (Applied Biosystems). Primer sequences for individual genes used in the Real-Time PCR assay are as follows: p16: 5′-CTTCGGCTGACTGGCTGG-3′ (forward) and 5′-TCATCATGACCTGGATCGGC-3′ (reverse), amplicon size: 129 bp; p21: 5′-TGCCGAAGTCAGTTCCTTGT-3′ (forward) and 5′-GTTCTGACATGGCGCCTCC-3′ (reverse), amplicon size: 86 bp; TFAM: 5′-CCAAAAAGACCTCGTTCAGCTT-3′ (forward) and 5′-CTTCAGCTTTTCCTGCGGTG-3′ (reverse), amplicon size: 87 bp; MFN1: 5′-AGTTGGAGCGGAGACTTAGC-3′ (forward) and 5′-ATCGCCTTCTTAGCCAGCAC-3′ (reverse), amplicon size: 76 bp; MFN2: 5′-AATCTGAGGCGACTGGTGAC-3′ (forward) and 5′-GGACATTGCGCTTCACCTTC-3′ (reverse), amplicon size: 126 bp; OPA1: 5′-TGCCTGACATTGTGTGGGAAA-3′ (forward) and 5′-TTCCGGAGAACCTGAGGTAA-3′ (reverse), amplicon size: 161 bp; SDHA 5′-TTGATGCAGTGGTGGTAGGC-3′ (forward) and 5′-TTGATTCCTCCCTGTGCTGC-3′ (reverse), amplicon size: 139 bp; SDHB 5′-GCAGCAGTATCTGCAGTCCA-3′ (forward) and 5′-CAGCGATAGGCCTGCATAAGA-3′ (reverse), amplicon size: 153 bp; SDHC 5′-GGAACCACGGCCAAAGAAGA-3′ (forward) and 5′-AGAGACCCCTGCACTCAAAG-3′ (reverse), amplicon size: 159 bp; SDHD 5′-ATACACTTGTCACCGAGCCAC-3′ (forward) and 5′-AAGGCCCCAGTGACCATGAAG-3′ (reverse), amplicon size: 177 bp; HK2: 5′- TTGAGAGCACCTGTGACGAC-3′ (forward) and 5′-CCACACCCACTGTCACTTTG-3′ (reverse), amplicon size: 156 bp; PFK1: 5′-TCCGACACAGTCTCCTGGAC-3′ (forward) and 5′- GCTGCCTCCTAGCGACTCTT-3′ (reverse), amplicon size: 187 bp; PKM1: 5’-CAGCACCTGATAGCTCGTGA-3′ (forward) and 5′-TTGAGGCTCGCACAAGTTCT-3′ (reverse), amplicon size: 76 bp; PDHA: 5’-CAGACTGTACGCCGAATGGA-3′ (forward) and 5′- AGCAAGCTTCCTGACCATCAC-3′ (reverse), amplicon size: 97 bp; PDHB: 5’- CTCCGGAAGCTCAGTCAAA-3′ (forward) and 5′-AGCACTGCTGCAGCTTCTAA-3′ (reverse), amplicon size: 130 bp; LDHA: 5’-ACGTCAGCATAGCTGTTCCA-3′ (forward) and 5′- TGGAACCAAAAGGAATCGGG-3′ (reverse), amplicon size: 139 bp; G6PD: 5’- CCCGGAAACGGTCGTACACT-3′ (forward) and 5′-CATGACGCTGTCTGCGCTTC-3′ (reverse), amplicon size: 72 bp; 18S: 5′-CCTTGGATGTGGTAGCCGTTT-3′ (forward) and 5′-AACTTTCGATGGTAGTCGCCG-3′ (reverse), amplicon size: 104 bp. PCR reactions were set up as follows: reverse transcriptase inactivation at 95 °C for 10 min followed by 40 cycles of 95 °C for 15 s and 60 °C for 1 min. The cycle threshold (C_T) was determined from the linear range of amplification and normalized to 18S rRNA levels for individual samples. The relative mRNA levels were calculated as follows: ΔC_T (sample) = C_T (mRNA of interest) - C_T (18S); relative expression = $2^{-\Delta\Delta CT}$.

siRNA knockdown

Human MFN1 (SR310844), OPA1 (SR303287), and scrambled (SR30004) siRNAs were purchased from OriGene. Confluent cultures of NHFs were transfected with siRNAs using Lipofactamine 2000 (Life Technologies). Quantitative RT-PCR measurements of MFN1 and OPA1 mRNA expression were performed to assess knock-down efficiency of MFN1 and OPA1 siRNAs.

Immunoblotting assay

Equal amounts of total protein lysates were separated on 12% sodium dodecyl sulfate polyacrylamide gel electrophoresis (SDS-PAGE) and electro-transferred to 0.45 μm nitrocellulose membrane (BioRad Labs). Immunoblotting was performed using antibodies to human MFN1 (1:1000, Abcam) and OPA1 (1:1000, BD Biosciences), SDHA (1:5000, Abcam), SDHB (1:200, Abcam), SDHD (1:1000, Millipore), PFKL (1:1000, Cell Signaling). Horseradish peroxidase conjugated secondary antibodies (anti-mouse: 1:5000; anti-rabbit: 1:5000, GE Healthcare), Pierce enhanced chemiluminescence Plus reagent (Thermo Scientific), and Typhoon FLA 7000 (GE Healthcare) were used for visualization of immune-reactive polypeptides. The blots were stripped and re-probed with actin (1:3000, Millipore) antibody for loading comparison. Images were quantitated using ImageJ software (National Institutes of Health). Fold change was calculated after correction for loading controls in each sample.

Metabolic flux analysis

Extracellular acidification rate (ECAR) and oxygen consumption rate (OCR) were measured using Seahorse XF96 Extracellular Flux Analyzer (Seahorse Bioscience). Contact inhibited quiescent cultures of NHFs were plated in Seahorse XF96 cell culture microplates at a density of 1.2×10^4 cells per well. Pre-warmed Seahorse XF assay media supplemented with 2 mM Glutamax pH 7.4 at 37 °C was used in the Glycolytic Stress Test and Seahorse XF assay media supplemented with 2 mM Glutamax, 1 mM sodium pyruvate, and 25 mM glucose pH 7.4 at 37 °C was used in the Cell Mito Stress test. The buffer capacity was determined before the experiment at 37 °C and pH 7.4 in both the Glycolysis Stress Test and the Cell Mito Stress Test (Divakaruni *et al.* 2014). Seahorse media addition were: glucose 10 mM, oligomycin 2.5 μM, 2-deoxyglucose (2-DG) 100 mM, carbonyl cyanide-4-(trifluoromethoxy)phenylhydrazone (FCCP) 0.5 μM, antimycin A 10 μM, and rotenone 10 μM final concentration. Cell number in individual wells at the end of the measurements was determined using a hemocytometer. ECAR is calculated as npH $cell^{-1}$ s^{-1} (pH units x 10^{-9} $cell^{-1}$ s^{-1}), and OCR results are presented as attomoles O_2 $cell^{-1}$ s^{-1} (abbreviation = amol O_2 $cell^{-1}$ s^{-1}). Lactate levels were measured using a Lactate Colorimetric/Fluorometric assay kit (BioVision Technologies). A CellTiter-Glo® Luminescent Cell Viability Assay kit (Promega) was used to measure cellular ATP content. Results are presented as femtomoles ATP per cell (fmol ATP $cell^{-1}$).

Imaging and quantitative analysis of mitochondria dynamics

Quiescent monolayer cultures of NHFs were incubated with Hanks buffered salt solution (HBSS) containing 0.5 μM MitoTracker Green (Invitrogen), and fluorescence was visualized (488 nm excitation laser and 530 nm emission filter) by using a Zeiss 510 confocal microscope (The University of Iowa Central Microscopy Research Core Facility). Images for single cells were captured using the Z stack function of 12 layered

images to compose a 3-dimensional image of the cells. Images were then processed and analyzed using Imaris Scientific 3D/4D Image Processing and Analysis Software (Bitplane Scientific Software). The Software creates a 3D image of the Z stack and then processes it to create a virtual model of the mitochondria network. The model was used to calculate the volume and number of compartments of the mitochondria network based on the connection and disconnection within the network. Results are plotted as total volume *vs.* compartment number ± SEM.

Senescence assay

Senescence-associated β-galactosidase Activity Assay (BioVision Technologies) was performed to measure cellular senescence. Senescence status was also examined by quantitative RT-PCR measurements of mRNA levels of cyclin dependent kinase inhibitors, p16 and p21.

PFK activity assay

PFK catalytic activity was measured using the Phophofructokinase Activity Colorimetric Assay Kit (BioVision Technologies). In presence of ATP, PFK converts fructose-6-phosphate to fructose-diphosphate and ADP. ADP is then converted to AMP and NADH. Measurements of NADH at 450 nm represent PFK activity.

MnSOD and CuZnSOD activity assay

Ten micrograms of total protein extract prepared from quiescent cultures of young and old NHFs were separated by native polyacrylamide gel electrophoresis (Darby Weydert *et al.* 2003). Gels were stained with nitro blue tetrazolium (NBT) and riboflavin-TEMED solution for 30 min at room temperature. The bands were visualized and quantified with a computerized digital imaging system interfaced with AlphaImager 2000 software (Alpha Innotech.). Results are representative of two or more experiments.

Complex II activity assay

A cell-based assay was used to measure Complex II activity (Zhang *et al.* 2013). NHFs were air-dried and incubated with 0.55 mM NBT and 0.05 M disodium succinate at 37 °C overnight. Cells were stained with Hoechst to visualize nuclei. Olympus CKX41 microscope (Olympus) and ImageJ software (National Institutes of Health) were used to quantitate results. The integrated density of each image was normalized to the number of nuclei in that field. Two thousand nuclei in each treatment group were analyzed, and fold change in complex II activity was calculated relative to complex II activity of 3-d NHFs.

Cell survival assay

A clonogenic assay was used to measure cell survival. Lethally irradiated (30 Gy) V79 Chinese Hamster Fibroblasts were used as a feeder layer for the clonogenic assay. Single cell suspension of control and irradiated NHFs were plated on top of the feeder layer and cultured for 14 d followed by fixation in ethanol and staining with 0.8%

Coomassie Brilliant Blue G-250 in 50% methanol and 20% acetic acid. Surviving fraction was calculated using the following formula: surviving fraction (SF) = (number of colonies counted) / (number of cells seeded × plating efficiency).

Statistical analysis

Statistical analyses were performed using GraphPad Prism software 6.0 (GraphPad Software Inc.). Statistical analysis was determined using the one-way analysis of variance (ANOVA) followed by Dunnett's post test; the Student's *t* test was used for experiments with less than three groups. Homogeneity of variance was assumed at 95% confidence interval. Results from at least three independent experiments with $p < 0.05$ were considered statistically significant.

SI FIGURE LEGENDS

Fig. S1. An age-related shift in metabolism from glycolysis in young to mitochondrial respiration in old NHFs is associated with: a decrease in lactate levels; an increase in ATP levels; an increase in cellular ROS levels; and a decrease in superoxide dismutase (SOD) and complex II activities. (A) A colorimetric assay (BioVision) was used to measure lactate levels. **(B)** CellTiter-Glo kit (Promega) was used to measure ATP levels. **(C)** Flow cytometry measurements of dihydroethidium (DHE) oxidation was used to assess cellular reactive oxygen species (ROS) levels. Asterisks represent statistical significance compared to 3-d NHFs; n = 3, $p < 0.05$. **(D)** A native gel electrophoresis assay was used to measure activities of MnSOD and CuZnSOD: representative nitroblue tetrazolium (NBT) stained gels are shown on left and quantitation of results are shown in the right. Results from the 3-d, 5-m, and 1-y NHFs were pooled as the Young group, and 58-y, 61-y, 63-y, 68-y, and 70-y NHFs were pooled as the old group. **(E)** A quantitative RT-PCR assay was used to measure mRNA levels of complex II subunits: succinate dehydrogenase A, B, C, and D. Asterisks represent statistical significance compared to mRNA levels of 3-d NHFs; n = 3, $p < 0.05$. **(F)** An immunoblotting assay was used to measure protein levels of complex II subunits. **(G)** A cell-based assay (Zhang *et al.* 2013) was used to measure complex II activity: representative microscopy images of cells showing complex II activity (purple color; magnification: x400) are shown on the left; cells with purple color were scored and normalized to the number of nuclei in each image using ImageJ software. A total of 2,000 nuclei in images from six different dishes were scored. Asterisks represent statistical significance compared to complex II activity of 3-d NHFs; n = 6, $p < 0.05$.

Fig. S2. Significant downregulation of phosphofructokinase 1 (PFK1) expression during aging: (A) A quantitative RT-PCR assay was used to measure mRNA expressions of HK2 (hexokinase 2), PFK1 (phosphofructokinase, muscle), PKM1 (pyruvate kinase, muscle), PDHA (pyruvate dehydrogenase alpha), PDHB (pyruvate dehydrogenase beta), LDHA (lactate dehydrogenase A), and G6PD (glucose-6-

phosphate dehydrogenase). Asterisks represent statistical significance compared to corresponding mRNA levels of 3-d NHFs; n = 3, $p < 0.05$. **(B)** A quantitative RT-PCR assay was used to measure PFK1 mRNA levels of 3-d, 12-y, and 61-y NHFs. Asterisks represent statistical significance compared to PFK1 mRNA levels of 3-d NHFs; n = 3, $p < 0.05$. **(C)** PFK1 mRNA levels of NHFs from the same individual at 29, 36, and 46 years of age. Asterisks represent statistical significance compared to 29-y NHFs; n = 3, $p < 0.05$. **(D)** Immunoblotting and **(E)** colorimetric (BioVision) assays were performed to measure PFK protein levels and activity, respectively. Asterisks represent statistical significance compared to PFK activity of 3-d NHFs; n = 3, $p < 0.05$.

Fig. S3. Perturbations in mitochondrial functions during aging: A Seahorse XF96 instrument was used to measure oxygen consumption rate (OCR) in quiescent cultures of NHFs from healthy donors of young (newborn, 3-d, 5-m, and 1-y) and old (58-y, 63-y, 68-y, and 70-y) individuals; **(A)** Basal respiration, **(B)** ATP-linked respiration, and **(C)** proton leak. Basal respiration was calculated after subtraction of non-mitochondrial respiration. ATP-linked respiration and respiration of proton leak was calculated following the addition of oligomycin. Asterisks represent significance compared to OCR of 3-d NHFs; n = 3, $p < 0.05$.

SI REFERENCES

Darby Weydert CJ, Smith BB, Xu L, Kregel KC, Ritchie JM, Davis CS, Oberley LW (2003). Inhibition of oral cancer cell growth by adenovirusMnSOD plus BCNU treatment. *Free radical biology & medicine*. **34**, 316-329.

Divakaruni AS, Paradyse A, Ferrick DA, Murphy AN, Jastroch M (2014). Analysis and interpretation of microplate-based oxygen consumption and pH data. *Methods in enzymology*. **547**, 309-354.

Zhang B, Davidson MM, Zhou H, Wang C, Walker WF, Hei TK (2013). Cytoplasmic irradiation results in mitochondrial dysfunction and DRP1-dependent mitochondrial fission. *Cancer research*. **73**, 6700-6710.

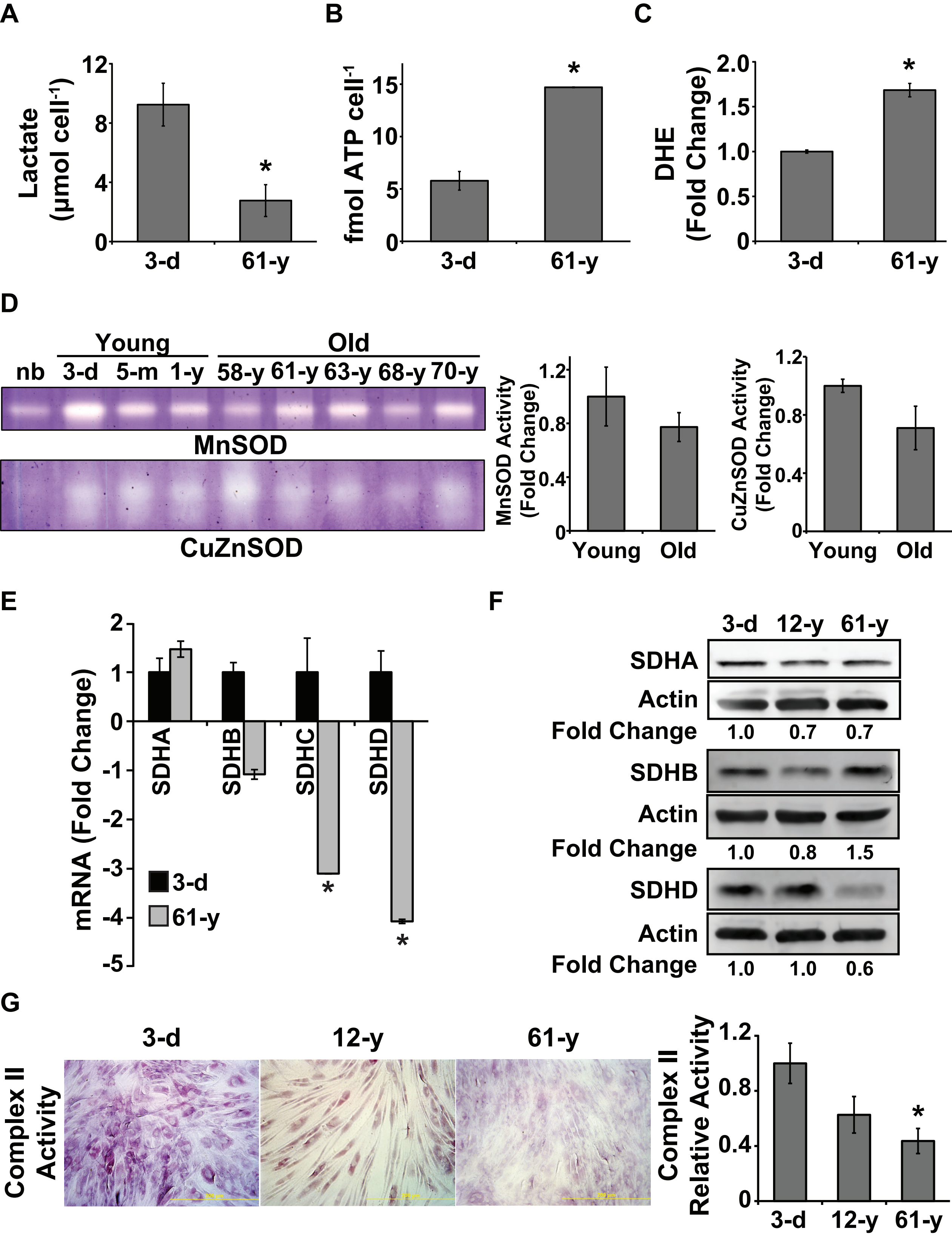

A
Lactate (μmol cell-1)
12
8
4
0
3-d
61-y
*
B
fmol ATP cell-1
15
10
5
0
3-d
61-y
*
C
DHE (Fold Change)
2.0
1.5
1.0
0.5
0
3-d
61-y
*
D
Young
Old
nb
3-d
5-m
1-y
58-y
61-y
63-y
68-y
70-y
MnSOD
CuZnSOD
MnSOD Activity (Fold Change)
1.2
0.8
0.4
0
Young
Old
CuZnSOD Activity (Fold Change)
1.2
0.8
0.4
0
Young
Old
E
mRNA (Fold Change)
2
1
0
-1
-2
-3
-4
-5
SDHA
SDHB
SDHC
SDHD
3-d
61-y
*
*
F
3-d
12-y
61-y
SDHA
Actin
Fold Change
1.0
0.7
0.7
SDHB
Actin
Fold Change
1.0
0.8
1.5
SDHD
Actin
Fold Change
1.0
1.0
0.6
G
3-d
12-y
61-y
Complex II Activity
Complex II Relative Activity
1.2
0.8
0.4
0
3-d
12-y
61-y
*

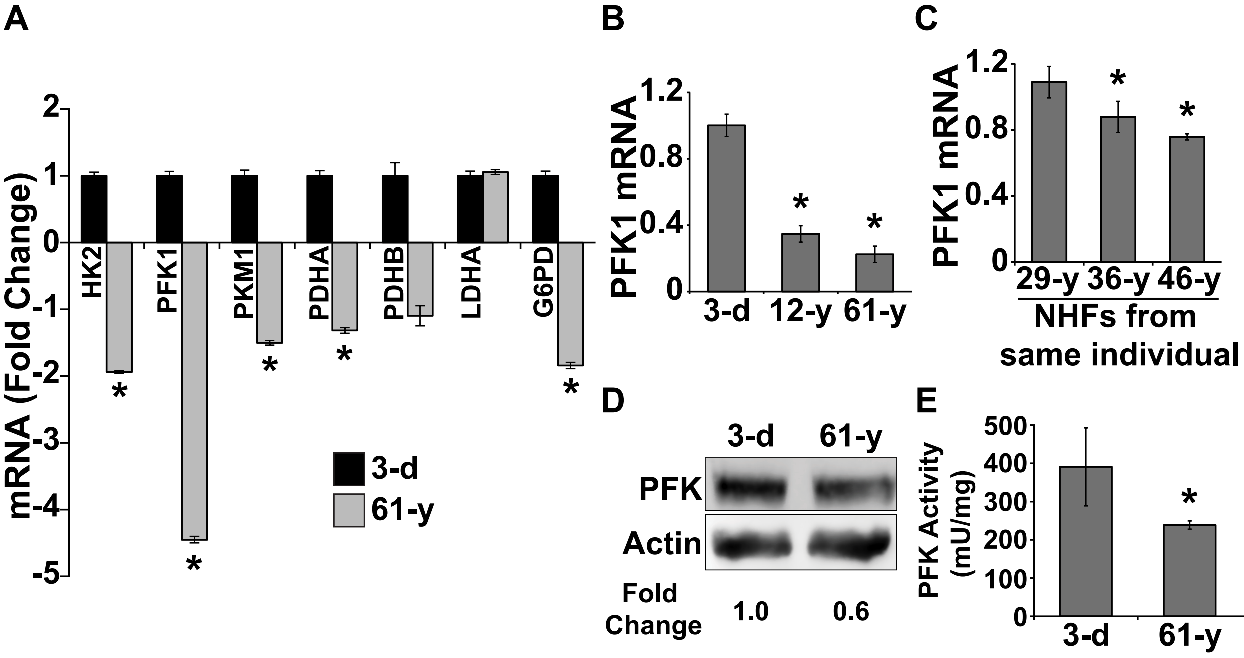

A
mRNA (Fold Change)
2
1
0
-1
-2
-3
-4
-5
HK2
PFK1
PKM1
PDHA
PDHB
LDHA
G6PD
*
3-d
61-y
B
PFK1 mRNA
1.2
0.8
0.4
0
3-d
12-y
61-y
C
PFK1 mRNA
1.2
0.8
0.4
0
29-y
36-y
46-y
NHFs from same individual
D
3-d
61-y
PFK
Actin
Fold Change
1.0
0.6
E
PFK Activity (mU/mg)
500
400
300
200
100
0
3-d
61-y

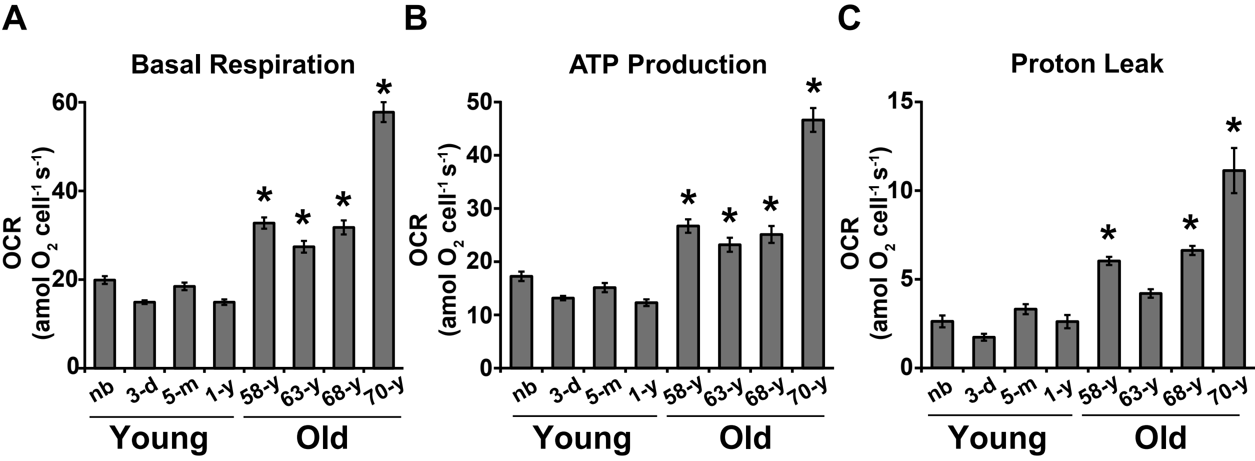
A
Basal Respiration
60
40
20
0
OCR
(amol O2 cell-1 s-1)
*
nb
3-d
5-m
1-y
58-y
63-y
68-y
70-y
Young
Old
B
ATP Production
50
40
30
20
10
0
OCR
(amol O2 cell-1 s-1)
nb
3-d
5-m
1-y
58-y
63-y
68-y
70-y
Young
Old
C
Proton Leak
15
10
5
0
OCR
(amol O2 cell-1 s-1)
nb
3-d
5-m
1-y
58-y
63-y
68-y
70-y
Young
Old
